# A PBMC-Based System to Assess Human T Cell Responses to Influenza Vaccine Candidates In Vitro

**DOI:** 10.3390/vaccines7040181

**Published:** 2019-11-13

**Authors:** Gabriela Tapia-Calle, Philip A. Born, Georgia Koutsoumpli, Martin Ignacio Gonzalez-Rodriguez, Wouter L. J. Hinrichs, Anke L. W. Huckriede

**Affiliations:** 1 Department of Medical Microbiology, University of Groningen, University Medical Center Groningen, Groningen, 9713AV Groningen, The Netherlands; gab.tapiac@gmail.com (G.T.-C.); g.koutsoumpli@umcg.nl (G.K.); martin.gonzalez.rodriguez@uta.fi (M.I.G.-R.); 2 Department of Pharmaceutical Technology and Biopharmacy, University of Groningen, 9713AV Groningen, The Netherlands; p.a.born@rug.nl (P.A.B.); w.l.j.hinrichs@rug.nl (W.L.J.H.)

**Keywords:** vaccines, in vitro, T cells, T follicular helper cells, influenza, WIV, split, peptides

## Abstract

Vaccine development is an expensive and time-consuming process that heavily relies on animal models. Yet, vaccine candidates that have previously succeeded in animal experiments often fail in clinical trials questioning the predictive value of animal models. Alternative assay systems that can add to the screening and evaluation of functional characteristics of vaccines in a human context before embarking on costly clinical trials are therefore urgently needed. In this study, we have established an in vitro system consisting of long-term cultures of unfractionated peripheral blood mononuclear cells (PBMCs) from healthy volunteers to assess (recall) T cell responses to vaccine candidates. We observed that different types of influenza vaccines (whole inactivated virus (WIV), split, and peptide vaccines) were all able to stimulate CD4 and CD8 T cell responses but to different extents in line with their reported in vivo properties. In-depth analyses of different T cell subsets revealed that the tested vaccines evoked mainly recall responses as indicated by the fact that the vast majority of the responding T cells had a memory phenotype. Furthermore, we observed vaccine-induced activation of T follicular helper cells, which are associated with the induction of humoral immune responses. Our results demonstrate the suitability of the established PBMC-based system for the in vitro evaluation of memory T cell responses to vaccines and the comparison of vaccine candidates in a human immune cell context. As such, it can help to bridge the gap between animal experiments and clinical trials and assist in the selection of promising vaccine candidates, at least for recall antigens.

## 1. Introduction

Vaccination is the primary measure for the control of infectious diseases and, in light of increasing resistance of microorganisms to antimicrobial treatment, might even gain more importance in the future. Yet, vaccine development is an expensive and time-consuming process with only a few successful outcomes. During vaccine development, monitoring vaccine immunogenicity represents a key step that, to date, essentially relies on animal testing. However, the predictive value of small animal models for the situation in humans is often poor due to intrinsic differences between the immune systems and very different immune biographies [1]. As a consequence, numerous vaccine candidates that succeeded in animal experiments subsequently performed poorly in clinical trials [2,3]. Frequent failure of vaccines during expensive clinical trials highlights the need for innovative experimental systems that can provide information about functional characteristics of vaccines in a human context, thus bridging the gap between the preclinical and clinical evaluation of vaccine candidates. 

Ideally, such systems would include different modules, allowing the assessment of vaccine effects on responses of antigen-presenting cells (APC) as well as T and B cells. We and others have previously established an APC module for determining the capacity of vaccines to induce innate immune responses [4,5]. Using this system, we demonstrated that primary human monocyte-derived dendritic cells (DCs) respond to different types of influenza vaccines in qualitatively and quantitatively characteristic ways, in line with the known in vivo properties of these vaccine types [4]. In the current paper, we focus on the T cell module, by evaluating an experimental system, which allows a detailed characterization of vaccine-evoked responses of human CD4^+^ and CD8^+^ T cells. 

To this end, we set up long-term cultures of unfractionated human peripheral blood mononuclear cells (PBMCs). As a proof of principle, we evaluated and characterized, in these cultures, (memory) T cell-mediated responses induced by two different well-characterized influenza vaccine formulations, H5N1-derived whole inactivated virus (WIV), and split virus, and further validated the model for a peptide-based influenza vaccine formulation. Activation, proliferation, increase of cytotoxic potential, and IFNγ responses were detected when the PBMCs were stimulated with WIV, split virus vaccine, or influenza peptides. Phenotyping of the CD4^+^ and CD8^+^ T cells revealed that the vaccines mainly evoked responses of effector memory and central memory T cell subsets, as expected. Interestingly, we could also identify vaccine-induced follicular T helper cell (T_FH_) responses. These data underscore the utility of the developed PBMC-based system to potentially bridge the gap between animal experiments and clinical trials and to assist in the selection of the most promising vaccine candidates for clinical evaluation, at least for recall antigens.

## 2. Materials and Methods 

### 2.1. Virus and Vaccines

The H5N1 virus (NIBRG-14, a 2:6 recombinant of A/Vietnam/1194/2004 (H5N1) and A/PR/8/34 (H1N1) virus produced by reverse genetics technology) was obtained from the National Institute for Biological Standards and Control, Potters Bar, United Kingdom. WIV and split vaccines were produced from this virus, as described before [4]. Briefly, H5N1 was propagated in embryonated chicken eggs and inactivated with 0.1% β-propiolactone for 24 h at 19–21°C, followed by dialysis and filtration (0.45 μm) to obtain WIV. A fraction of the WIV vaccine was then used to produce a split virus vaccine, as previously described [6]. In short, Triton X-100 was added to WIV in the presence of Tween 80, and the suspension was stirred for 1 h at 20 °C. Detergents were removed by overnight rotation of the vaccine together with Biobeads (Bio-Rad, Hercules, CA, USA) at 4°C. Vaccines were then characterized by SDS-PAGE, followed by silver staining. Protein content was determined by Lowry assay.

### 2.2. Peptides 

From a previous publication [7], we selected four different peptides representing 2 CD4 and 2 CD8 influenza epitopes. The peptides (lyophilized acetate salt; >98.0% purity, Caslo ApS, Lyngby, Denmark) were derived from the NP and the M protein of influenza A H3N2 virus and were highly conserved across a range of influenza virus strains. The peptides were selected such that they could be presented by several MHC haplotypes (Appendix A Table A1). A commercial peptide mixture consisting of 23 HLA class I-restricted T cell epitopes from cytomegalovirus, Epstein-Barr virus, and influenza virus (CEF pool standard; JPT, Berlin, Germany) was used as the positive control. 

### 2.3. Human Primary Cells

Human PBMCs were isolated from buffy coats from healthy volunteers (with unknown vaccination status) obtained from the Dutch blood bank (Sanquin, Nijmegen, The Netherlands). PBMCs were isolated by density gradient using Ficoll Paque (GE Healthcare, Uppsala, Sweden), as previously described [4]. PBMCs were stored in liquid nitrogen until needed. 

### 2.4. In Vitro Stimulation Using Influenza Vaccines and Peptides

PBMCs were thawed in a water bath at 37°C, as previously described [4]. Cells were seeded at a concentration of 1 × 10^6^/mL in 24-well plates in RPMI-1640 (l-glutamine and sodium bicarbonate) (Sigma Aldrich, St. Louis, MO, USA) supplemented with 10% FCS, 50 µM 2-mercaptoethanol, and 1% penicillin/streptomycin. Cells were incubated at 37°C, 5% CO_2_. After resting of the cells for 24 h, the different compounds (either WIV (10 μg/mL corresponding to viral hemagglutinin (HA), split (10 μg/mL corresponding to HA), influenza peptides (2.5 nmol/mL), or CEF pool (2 μg/mL)) were added and remained with the cells for the rest of the culture period. On day 5, 50% of the medium was replaced with fresh medium, and the cells were cultured for an additional 5 days. For cells to be analyzed by flow cytometry, Brefeldin A (eBioscience, San Diego, CA, USA) was added 12 h prior to harvesting. 

### 2.5. Multiparameter Flow Cytometry

On day 10, Brefelding-treated cells were harvested and washed using FACS buffer (1X PBS supplemented with 2% FCS and 1 mM EDTA), followed by staining with a fixable viability dye (Viobility 405/450, Miltenyi Biotec, Bergisch Gladbach, Germany) for 15 min at room temperature. Cells were then fixed and permeabilized using the BD Cytofix/Cytoperm Kit (BD Biosciences). Briefly, cells were incubated in the Fixation/Permeabilization solution for 20 min at 4°C, followed by the addition of BD Perm/Wash Buffer (1X). Cells were then incubated with both solutions for an additional 15 min and then centrifuged at 350 g for 5 min (Allegra X-15R, SX 4750/SX4750A Beckman Coulter; Brea, CA, USA). Intracellular staining with anti-IFNγ, or anti-IL21 and anti-IL12 (Miltenyi Biotech, Bergisch Gladbach, Germany), was performed, followed by surface staining with antibodies to CD3, CD4, CD8, CD25, CD107, CCR7, CD45RO, CXCR5, and ICOS (Appendix A Table A2) (all from Miltenyi Biotec, Bergisch Gladbach, Germany) according to the manufacturer’s instructions. For the proliferation assays, on day 0, cells were incubated with 2.5 μM carboxyfluorescein succinimidyl ester (CFSE) for 8 min, washed 3 times with CFSE buffer (PBS, 10% FBS), and seeded as mentioned above. Cells were acquired using a FACSVerse (BD Bioscience, San Jose, CA, USA), and data were analyzed using FlowLogic (Miltenyi Biotec, Bergisch Gladbach, Germany). 

### 2.6. ELISpot

On day 9, MultiScreen HTS IP filter plates (MabTech, Stockholm, Sweden) were coated with 5 μg/mL of IFNγ mAB 1-DK (MabTech, Stockholm, Sweden) in PBS and incubated overnight at 4°C. After 24 h, plates were washed with 1X PBS and incubated with 100 μL of X-VIVO 15 medium (Lonza) for an hour at 37°C with 5% CO_2_. Treated PBMCs were harvested, counted, and resuspended in an X-VIVO 15 medium to be seeded in triplicate at a concentration of 3 × 10^5^/100 μL. After seeding, cells were re-stimulated with either WIV or split vaccine (10 µg/mL HA), influenza peptides (1.25 nmol/mL), or CEF (2 µg/mL pool) and incubated overnight at 37°C, 5% CO_2_. Twenty-four hours after stimulation, plates were thoroughly washed with PBS/Tween 0.05%, followed by incubation with mAb 7-B6-1-biotin (1:3000, Mabtech, Stockholm, Sweden) for 2 h at room temperature in the dark. Plates where then washed and incubated with extravidin-alkaline phosphatase (ALP) (1:1000) for 1 h at room temperature. Finally, plates were washed and developed (substrate solution: BCIP/NBT ALP in miliQ water) for 7 min. The frequency of IFN-γ producing cells was measured using an AID ELISpot/Fluorospot reader and analyzed with the AID ELISpot 6.0 iSpot software (Strassberg, Germany).

### 2.7. Statistical Analysis

Comparisons were performed using the Wilcoxon matched-pair Ƭ test, assuming a non-Gaussian distribution and a 95% confidence level or using the Friedman test with post hoc Dunnett’s multiple comparison test. A *p*-value < 0.05 was considered significant and is indicated by *, ** stand for 0.01 and *** for 0.001. Statistical analyses were performed with GraphPad Prism version 7.0 (GraphPad Software, San Diego, CA, USA). 

## 3. Results

### 3.1. Antigen-Specific T Cells Expand Upon In Vitro Stimulation with WIV Vaccines

**Figure 1:**
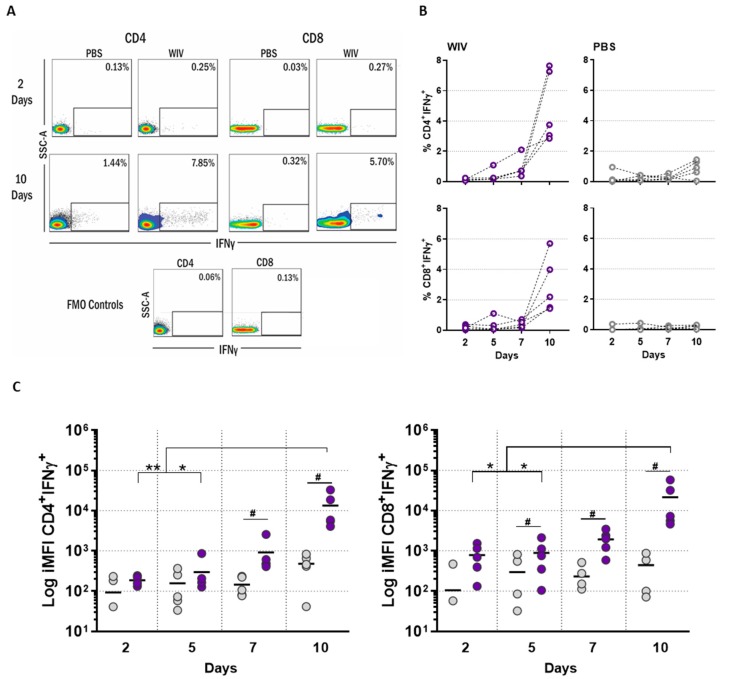
The frequency of antigen-specific T cells and the total amount of IFNγ produced increase over time. Peripheral blood mononuclear cells (PBMCs) were stimulated with PBS or whole inactivated virus (WIV) over a 10-day period. Cells were harvested at different time points after stimulation and evaluated for the production of IFNγ by CD4^+^ and CD8^+^ T cells using flow cytometry. (**A**) Representative dot plots depicting the expression of IFNγ by CD4^+^ and CD8^+^ T cells in PBMCs stimulated with WIV (purple) or PBS (grey) for 2 or 10 days (gating as in Appendix A Figure A1). (**B**) Percentages of IFNγ-positive CD4^+^ and CD8^+^ T cells at the indicated days. (**C**) iMFI of IFNγ for PBS (grey) and WIV (purple) stimulated cells for CD4^+^ and CD8^+^ T cells. Each symbol represents a donor (*n* = 5). Asterisks indicate statistically significant differences between days, and hashes indicate statistically significant differences to PBS. *p* < 0.05 = * and ** <0.01. *p* < 0.05 = #.

**Figure A1:**
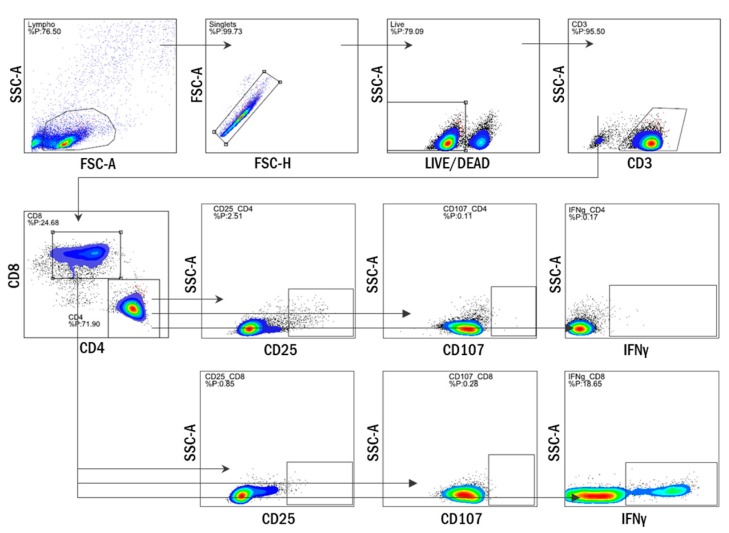
Gating strategy for IFNγ, CD25, and CD107 in CD4^+^ and CD8^+^ T cell subsets. Overnight rested-PBMCs were stimulated with WIV, split, CEF, or PBS-treated for 10 days. A seven-color flow cytometry panel was used to assess the expression of CD25, CD107, and IFNγ in the CD4^+^ and CD8^+^ T cells.

To establish a PBMC-based system to assess vaccine-induced T cell responses in vitro, we first set out to understand the kinetics of the T cell responses over time. Hence, freshly thawed unfractionated PBMCs were rested overnight and stimulated from day one onwards with the H5N1-derived WIV influenza vaccine or mock-stimulated with PBS. At different time points, the cells were harvested for intracellular staining of IFNγ followed by flow cytometric analysis (see Appendix A Figure A1 for gating strategy). In WIV-treated PBMC cultures, influenza-specific T cells were detected from day two onwards, although initially at low frequency, confirming the presence of influenza-specific T cells in our donors. Overall, the percentages of IFNγ-producing T cells in WIV-treated cell cultures remained low until day seven but thereafter increased ~2- to 6-fold until day 10 for both CD4^+^ and CD8^+^ T cells (Figure 1A,B). No expansion was observed in PBS-treated cell cultures.

To get a better picture of the total amount of IFNγ produced per T cell subtype, we calculated the integrated median fluorescence intensity (iMFI) as the product of cell frequency and median fluorescence intensity (MFI). As previously stated, the iMFI depicts the total functional response of a given cytokine [8]. Already by day two, we observed that CD8^+^ T cells produced higher amounts of IFNγ in WIV-stimulated than in mock-treated PBMC cultures (Figure 1C). On subsequent days, the amount of IFNγ generated (iMFI) increased in WIV-stimulated cultures and was significantly higher than in PBS-treated PBMCs for both T cell populations from day seven onwards. On day 10, the total amount of IFNγ in CD4^+^ and CD8^+^ T cells in WIV-treated PBMCs was significantly higher than on days two and five (Figure 1C). In contrast, the total amount of IFNγ produced by PBS-treated cells remained similar throughout the experiment.

**Figure A2:**
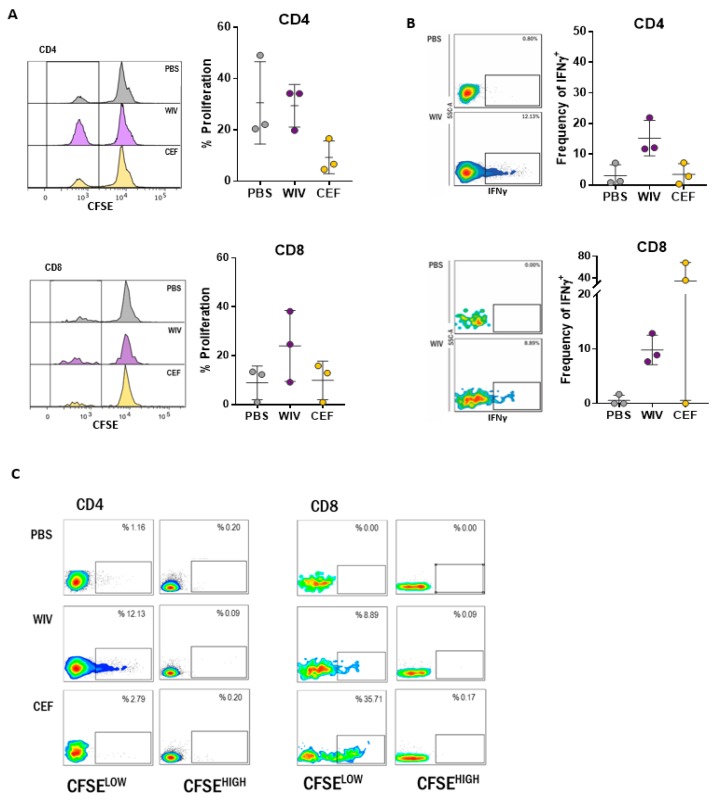
WIV induces the expansion of IFNγ-producing CD4^+^ and CD8^+^ T cells. CFSE-treated PBMCs were incubated with WIV (purple), CEF (positive control, yellow), or PBS (negative control, grey) for 10 days. (**A**) Representative proliferation histograms (left) and percentage of proliferating cells in the investigated donors (right). (**B**) Representative histograms showing the gating of the IFNγ^+^ population in the proliferating fraction for WIV and PBS-treated PBMCs (left). Percentage of IFNγ^+^ CD4^+^ and CD8^+^ T cells in the proliferating fraction (right) (*n* = 3). (**C**) Representative plots depicting the induction of IFNγ in the CFSE^LOW^ (proliferating) and the CFSE^HIGH^ (non-proliferating) population.

To determine whether the observed increase in frequency of IFNγ-producing T cells in WIV-treated PBMC cultures was due to proliferation, PBMCs were labeled with CFSE and exposed to WIV, CEF pool (positive control for CD8 stimulation), or PBS for 10 days and analyzed by flow cytometry. The proliferation of CD4 T cells was observed for all conditions but was stronger in the WIV- and PBS-treated than in the CEF-treated cultures (Appendix A Figure A2A). However, only the WIV-treated and not the PBS- or CEF-treated PBMCs showed the production of IFNγ and only in the proliferating (CFSE^LOW^) fraction (Appendix A Figure A2B). In the CD8^+^ subset, WIV induced stronger proliferation than CEF and PBS. As in the CD4^+^ T cell subset, only cells stimulated with WIV (and CEF) produced IFNγ and IFNγ production was restricted to the proliferating fraction (Appendix A Figure A2C).

These results corroborated that influenza-specific responses can be detected in PBMCs from healthy individuals after two days of stimulation with WIV, as expected. The culture of unfractionated PBMCs with WIV for a 10-day period enabled the expansion of, most probably, pre-existing, antigen-specific CD4^+^ and CD8^+^ T cells. The total IFNγ response, defined as iMFI, increased by a factor of 100 in both T cell populations. Given this observation, we decided to focus on day 10 for the following experiments. 

### 3.2. T Cell Responses in Long-Term PBMC Cultures Are Vaccine Formulation-Specific

**Figure 2:**
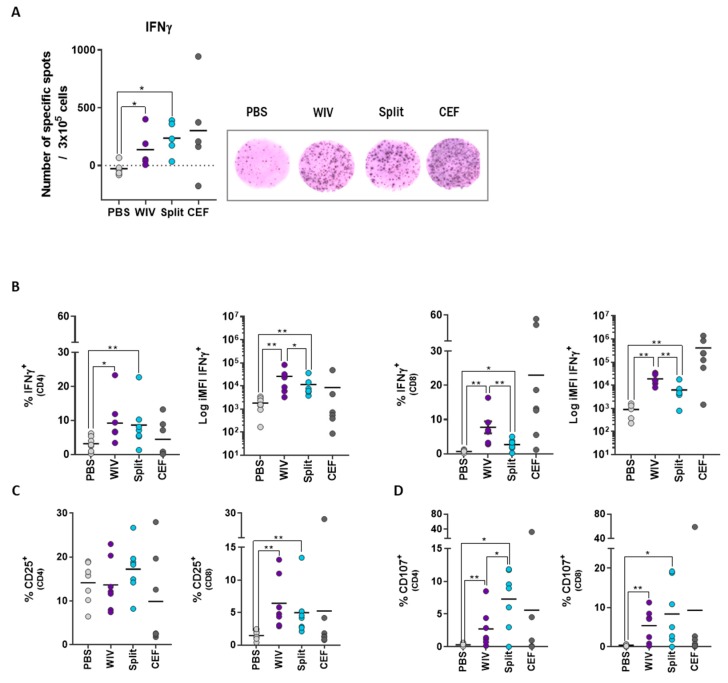
WIV and split vaccine induce the production of IFNγ, activation, and cytotoxic potential in CD4^+^ and CD8^+^ T cells. Human PBMCs were stimulated with WIV (purple) or split (blue) influenza vaccine formulations. After 10 days of culture, cells were evaluated by ELISpot and multicolor flow cytometry. (**A**) For the ELISpot, cells were harvested at day 10, plated, and re-stimulated overnight. Depicted are the numbers of specific spots/3 × 10^5^ cells, which represent the normalized data after subtracting the PBS control using the ELISpot assays (*n* = 5). (**B**) Harvested cells were stained for multicolor flow cytometry. Depicted are the percentages and the iMFIs of IFNγ^+^ cells, (**C**) the percentages of CD25^+^ cells, and (**D**) CD107^+^ cells (*n* = 7). CEF (dark grey) and PBS (light grey) were used as internal positive and negative controls (respectively) for each individual. Asterisks indicate statistical significance, as explained in the legend to Figure 1. *p* < 0.05 = * and ** <0.01.

We next determined whether the T cells in our in vitro system would respond differently to different types of vaccines. For this purpose, we used two different influenza vaccine formulations; WIV and split. These vaccines have the same protein content but differ in their stimulatory capacity, as WIV contains RNA capable of signaling through Toll-like receptor 7 (TLR7) while split does not [9]. WIV particles are also more easily taken up by APCs than split, which consists of solubilized particles [10]. Furthermore, WIV retains membrane fusion properties, thus favoring CTL responses [11]. We first performed an ELISpot assay, which is considered to be more sensitive for the detection of antigen-specific T cells than intracellular cytokine staining (ICS) [12] but does not allow to discriminate between CD4- and CD8- derived cytokines. After ten days of culture, we observed that the PBMCs responded equally well to both vaccines by displaying high numbers of IFNγ-producing cells. Only a few background IFNγ-producing cells were observed after treatment with PBS (Figure 2A).

Having established the antigen-specific responses by ELISpot assay, we characterized the responding T cells in more detail using ICS and multicolor flow cytometry. We observed that treatment with both vaccines led to a significantly higher number (%) of IFNγ-producing CD4^+^ and CD8^+^ T cells and to a higher total amount (iMFI) of IFNγ than treatment with PBS. The total amount of IFNγ (iMFI) induced in the CD4^+^ and the CD8^+^ T cells by WIV stimulation was significantly higher than that elicited by the split vaccine (Figure 2B). Next, we assessed the activation profile of the cells by checking the expression of the interleukin (IL-) 2 receptor α-subunit (CD25), a late activation marker [13]. We observed that exposure to both WIV and split vaccines resulted in higher percentages of CD8^+^CD25^+^ T cells than observed in PBS-treated cultures, while it did not affect the percentages of CD4^+^CD25^+^ T cells (Figure 2C). Additionally, we determined the cytotoxic potential of T lymphocytes by staining for the lysosome-associated membrane protein LAMP-1 (CD107) [14]. Intriguingly, both vaccines elicited upregulation of this marker not only in CD8^+^ but also in CD4^+^ cells, demonstrated by significantly higher percentages of CD107^+^ cells than in PBS-treated cultures (Figure 2D). Unexpectedly, the split vaccine induced significantly higher percentages of CD4^+^CD107^+^ than WIV. 

Altogether, these observations highlight the suitability of the used in vitro PBMC-based platform for a detailed characterization of antigen-specific responses to different influenza vaccine formulations.

### 3.3. Memory T Cell Subsets Are the Main Source of Antigen-Specific Responses In Vitro

**Figure A3:**
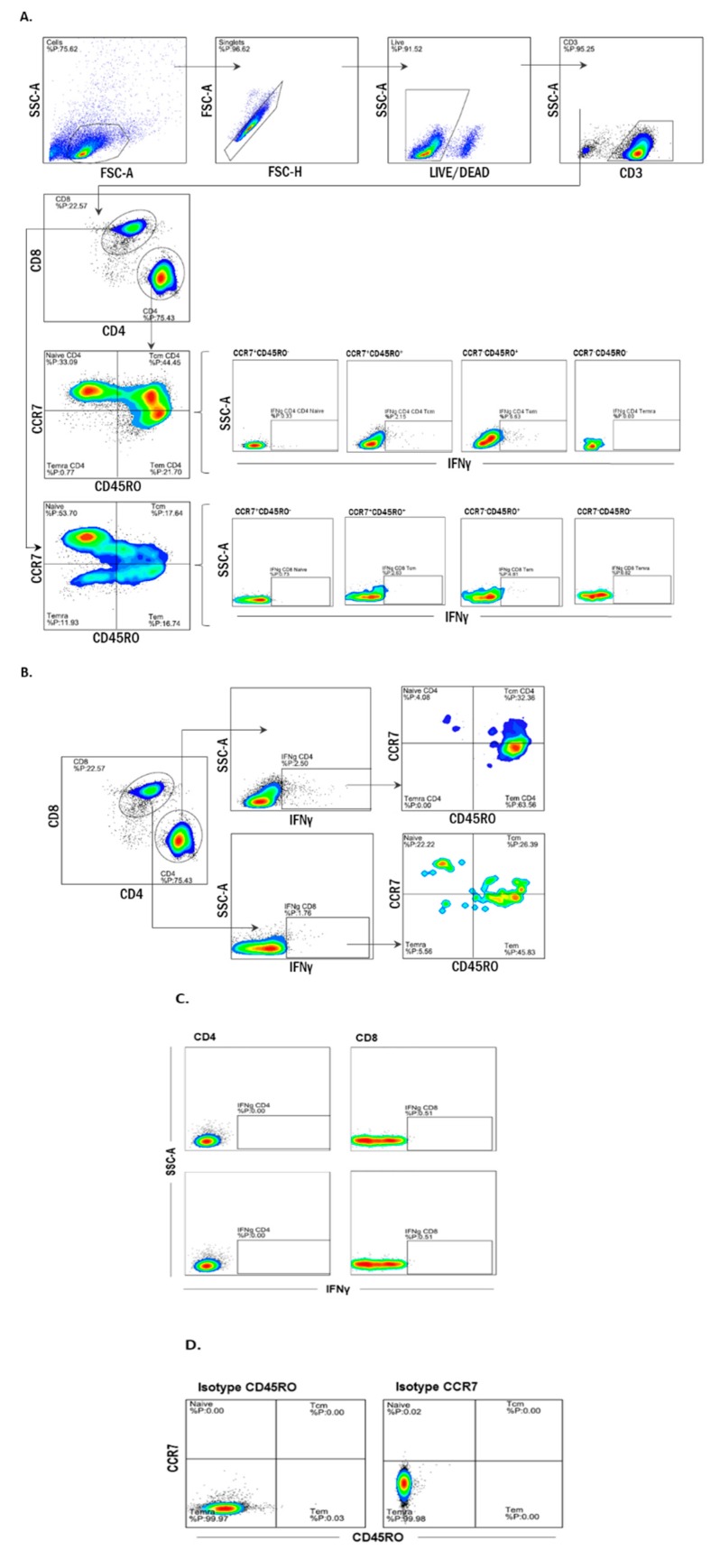
Gating strategies for the discrimination of different T cell subsets and their production of IFNγ. (**A**) A seven-color flow cytometry panel was used to assess the expression of CCR7, CD45RO, and IFNγ in human PBMCs. (**B**) Backgating analysis; starting from the IFNγ^+^ cells, cells were phenotyped based on their expression of CD45RO and CCR7. (**C**) FMO controls for the discrimination of IFN^+^ responses. (**D**) CCR7 and CD45RO isotype controls to define the different T cell naïve and memory subsets.

Classification of T cells into different memory subsets has been extensively used to understand the functional potential of these cells, especially in the context of vaccination [15,16,17]. On the basis of the expression of CCR7 and CD45RO, the following subsets are distinguished [18]: naïve (CD45RO^-^CCR7^+^), central memory (T_CM_: CD45RO^+^CCR7^+^), effector memory (T_EM_: CD45RO^+^CCR7^−^), and terminally differentiated (T_EMRA_: CD45RO^-^CCR7^−^) T cells (gating strategy depicted in Appendix A Figure A3A). In order to better understand the T cell responses evoked by exposure to WIV and split, we checked the induction of antigen-specific responses in each subset by flow cytometry.

**Figure 3:**
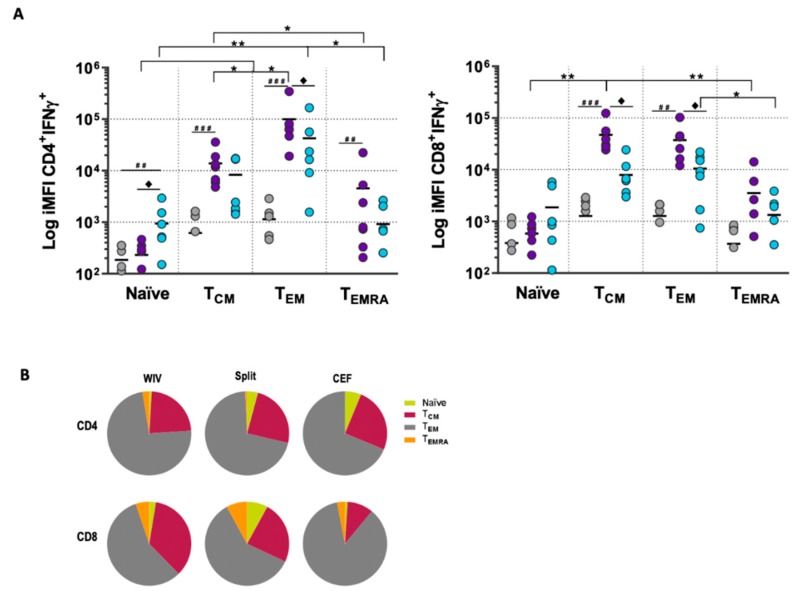
WIV and split vaccines induce the production of IFNγ by central memory and effector memory T cells. (**A**) Vaccine-stimulated PBMCs were phenotyped into naïve, T_CM_, T_EM_, T_EMRA_ by staining for CCR7 and CD45RO, and the production of IFNγ in each subpopulation was assessed by ICS (for gating strategy see Appendix A Figure A3A). Depicted are the iMFIs indicating the total production of IFNγ induced by WIV (purple), split (blue), or PBS (grey) in each T cell subset for each donor (*n* = 7). Hashes (#) indicate statistical significance compared to PBS-treated cultures, diamonds (♦) indicate the statistical difference between WIV and split vaccines, and asterisks (*) indicate the statistical difference between the subsets. For the CD4 T cells, the log iMFI of T_EMRA_ was below 10 and is therefore not visible. (**B**) Pie chart of a representative donor depicting the contribution of naïve, T_CM_, T_EM_, and T_EMRA_ to IFNγ production as determined by back-gating of the IFNγ^+^ population (by assessing the expression of CCR7 and CD45RO of IFNγ^+^ cells, see Appendix A Figure A3B). Each pie represents a single condition (WIV, split, or CEF) for one donor. *p* < 0.05 = *, ** <0.01 and *** <0.001. *p* < 0.05 = #, ## <0.01 and ### <0.001.

Naïve T cells had only a minor contribution to the total IFNγ response, although the amount of IFNγ produced by naïve CD4^+^ cells differed significantly between split and PBS-treated PBMCs (Figure 3A). Stimulation with both vaccines led to significantly higher amounts of total IFNγ in all three CD4^+^ memory subsets (T_CM_, T_EM_, T_EMRA_) and two of the CD8^+^ memory subsets (T_CM_, T_EM_) than observed in the PBS controls. Across all memory T cell subsets, there was a consistently higher production of IFNγ in WIV-stimulated than in split-stimulated PBMCs; this difference was significant for CD4 T_EM_, CD8 T_EM_, and CD8 T_CM_. (Figure 3A). To further discriminate and quantify the contribution from each of the memory subsets to the antigen-specific responses, we performed a back-gating analysis in which we phenotyped—based on CCR7 and CD45RO expression—the IFNγ^+^ population (gated as in Appendix A Figure A3B). This enabled us to determine to which T cell subset the IFNγ^+^ cells allocated. In both the CD4^+^ and CD8^+^ T cell populations, the T_EM_ subset contributed most strongly to the production of IFNγ, followed by the T_CM_ subset. The subset allocation of the IFNγ-producing T cells was independent of the stimulus (WIV, split, or CEF) used. 

Overall, these experiments led us to conclude that memory T cells, specifically CD4 T_EM_, were primarily responsible for the antigen-specific responses seen upon in vitro stimulation with WIV and split vaccines. 

### 3.4. PBMCs Display Antigen-Specific Responses upon Stimulation with Influenza Peptides

**Figure 4:**
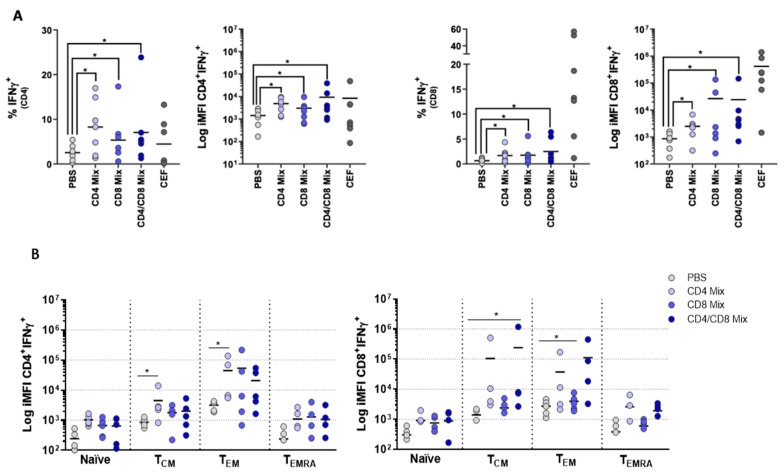
Peptides derived from influenza virus proteins stimulate the production of IFNγ by central memory and effector memory T cell subsets. PBMCs were stimulated with different combinations of influenza peptides targeting CD4^+^ or CD8^+^ T cells or with a mix of both. (**A**) Ten days after culture, cells were assessed for the expression of IFNγ, depicted are the percentages of the cells expressing IFNγ or the iMFIs for CD4^+^ and C8^+^ T cells (*n* = 7). (**B**) Production of IFNγ by each T cell subset (naïve, T_CM_, T_EM_, T_EMRA_) was assessed by determining the iMFI. Each symbol represents one donor. Significance was assessed by comparing each condition to the PBS control (*n* = 5). *p* < 0.05 = *.

In order to validate our approach to assess vaccines in vitro, we determined the induction of antigen-specific responses by peptide influenza vaccine candidates. For this purpose, we selected, from a previous study by Wilkinson et al. [7], two peptides representing CD4 epitopes and two peptides representing CD8 epitopes. For the peptide selection, we looked for those epitopes conserved across different influenza strains and to which responses were most frequently found in the human population. We first investigated whether these peptides could induce any IFNγ production. To this end, PBMCs were stimulated either with CD4 peptides (CD4 Mix), CD8 peptides (CD8 Mix), or a combination of the peptides (CD4/CD8 Mix). PBMCs stimulated with either of the peptide mixes or with CEF as a positive control contained significantly higher percentages of IFNγ^+^ CD4 and CD8 T cells than found in PBS-treated controls (Figure 4A). The CD4 peptide mix stimulated CD4^+^ but also CD8^+^ T cells, and also, the CD8 peptide mix stimulated both cell populations. This was in line with the in silico analysis of MHC binding (using SYFPEITHI), which predicted the promiscuity of the peptides that the CD4 peptides could potentially bind to MHC-I molecules and the CD8 peptides to MHC-II molecules. 

We additionally characterized the T cell subsets involved in the antigen-specific responses. Similar to that found for WIV and split vaccines, either of the mixes induced minor responses of the naïve T cell compartment and the T_CM_ and T_EM_ subsets were the main contributors to IFNγ production (Figure 4B). In addition, we observed higher donor-to-donor variation in response to the peptides than to WIV and split vaccines, as is to be expected due to the heterogeneity in HLA haplotypes in the population. Taken together, the fact that the T cells readily responded to peptide vaccine candidates confirms the versatility and robustness of our in vitro approach to assess antigen-specific T cell reactions. 

### 3.5. Induction of Follicular T Helper Cells by Influenza Vaccines In Vitro 

Recent studies aiming at unraveling the role of T_FH_ cells in the context of vaccination have shown that expansion of this subset in blood after vaccination can predict the titer of neutralizing antibodies at later time points [19]. Given this, we set out to assess whether we could detect the expansion and activation of these cells in our in vitro approach. We identified T_FH_ cells as CD4^+^CXCR5^+^ lymphocytes and assessed their activation status by measuring the expression of inducible T-cell co-stimulator (ICOS) alone or in combination with IL-21 by flow cytometry [19,20,21,22]. 

**Figure 5:**
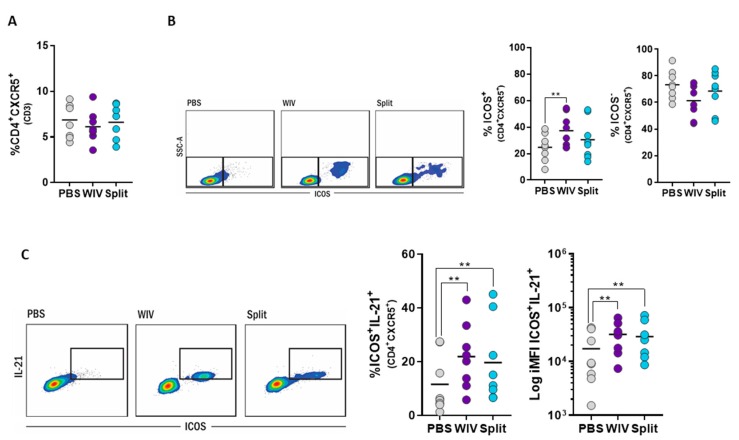
WIV and split vaccines induce the activation and the production of IL-21 in T_FH_ cells. Human PBMCs were stimulated with WIV (purple), split (blue), and PBS-treated (grey) for 10 days. Cells were harvested and analyzed with flow cytometry to assess the induction of T_FH_ cells. (**A**) Dot plots show the percentage of T_FH_ (CD4^+^CXCR5^+^) cells for each condition. (**B**) Representative flow cytometry plots depict the activated (ICOS^+^) and non-activated (ICOS^−^) T_FH_ populations (CD4^+^CXCR5^+^) induced by 10-day exposure to PBS, WIV, or split. The following dot plots show the percentages of activated and non-activated T_FH_ in all the donors tested. (**C**) Flow cytometry plots representative of the induction of IL-21 upon treatment with the different vaccines. Dot plots depict the percentage of cells expressing IL-21 and the iMFI of the respective population. *p* < 0.05 = * and ** <0.01.

**Figure A4:**
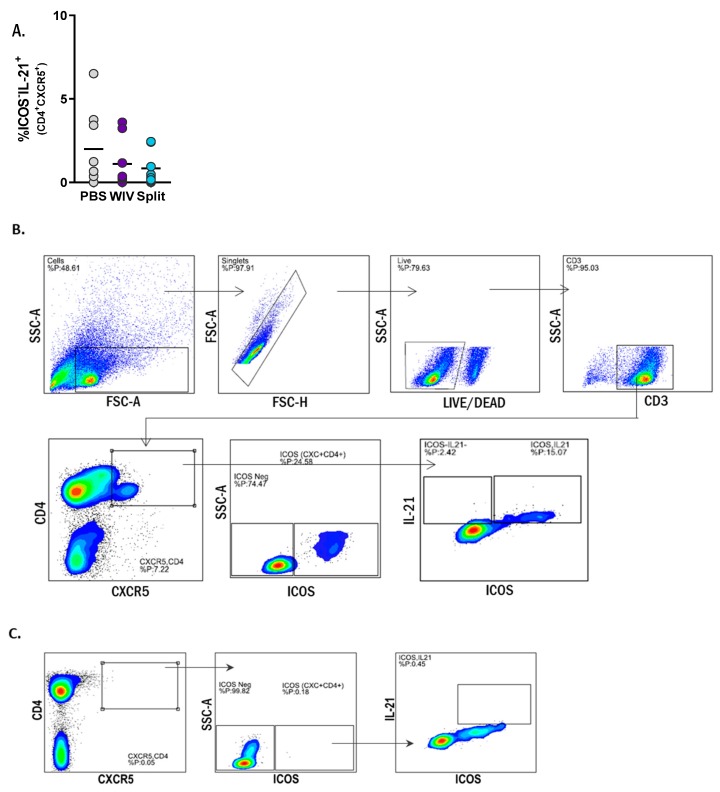
(**A**) Non-activated T_FH_ cells (ICOS^-^CD4^+^CXCR5^+^) do not produce IL-21. T_FH_ that displayed a non-activated phenotype (ICOS^−^) were also not able to produce IL-21. (**B**) Gating strategy for the discrimination of T_FH_ cells. A six-color flow cytometry panel was used to assess the expression of CXCR5, ICOS and IL-21 in human PBMCs. (**C**) FMO controls were used to set the gating strategy to define activated- and IL-21-producing T_FH_ cells.

After 10 days of in vitro culture, PBMC cultures stimulated with WIV or split vaccine contained similar frequencies of T_FH_ cells (CD4^+^CXCR5^+^) as found in mock-stimulated controls (Figure 5A). However, in WIV-treated PBMCs, a significantly higher percentage of the T_FH_ cells displayed an activated phenotype (ICOS^+^CD4^+^CXCR5^+^) than in mock-stimulated PBMCs (Figure 5B). Accordingly, the percentages of ICOS^-^CD4^+^CXCR5^+^ T cells were lower in WIV- than in PBS-treated cells. For the split vaccine, an increase in activated (ICOS^+^) T_FH_ was also observed, but this was not significant. Further zooming in on the activated T_FH_ population revealed that many of these cells were able to produce IL-21 (Figure 5C). In contrast, non-activated (ICOS^-^) T_FH_ cells were not able to produce this cytokine (Appendix A Figure A4). Thus, activation was required for cytokine production and was achieved by stimulation with WIV and, to a lower extent, by stimulation with the split vaccine. 

Altogether, these results show that T_FH_ cells can be readily identified in long-term cultures of unfractionated PBMCs and can be activated by stimulation with appropriate vaccines. Since activated T_FH_ cells are the key to B cell stimulation [23,24,25,26], this result highlights the possibility of using long-term PBMC cultures not only to assess T cell responses but also to predict vaccine immunogenicity in terms of antibody responses. 

## 4. Discussion

In this study, we examined whether long-term cultures of unfractionated PBMCs are suitable to assess the effects of H5N1 influenza vaccines on human T cells. By using a multicolor flow cytometry approach, we observed that: (1) stimulation of long-term cultures with WIV induced T cell expansion and the production of IFNγ; (2) T cells in long-term cultures responded to WIV, split, and peptide vaccines in quantitatively distinct ways with responses to WIV being most pronounced; (3) responses were derived mainly from memory subsets, T_EM_ for both CD4^+^ and CD8^+^ T cells; (4) vaccines enhanced the number of activated (ICOS^+^) T_FH_ and activated T_FH_ producing IL-21. These results demonstrate the potential suitability of this long-term culture in vitro system for assessing the effects of vaccines and vaccine candidates on human memory T cells.

Traditional culture systems for T cells either aim at determining antigen-specific memory responses induced by a previous infection or vaccination or attempt to induce or expand antigen-specific T cells in vitro [27,28,29,30,31]. For the determination of memory T cell responses, cells are harvested from blood, stimulated for a short period of time with antigenic peptides or other stimuli, and responses are assessed by techniques like proliferation assay, ELISpot assay, or intracellular cytokine staining. Experimental systems for in vitro induction or expansion of antigen-specific T cells usually make use of co-cultures of isolated DCs and autologous T cells [27,28,29,31,32]. However, this is a laborious approach that involves isolation of monocytes and T cells from peripheral blood, differentiation of monocytes to DCs, loading of DCs with antigen, and the combination of antigen-loaded DCs with T cells derived from frozen PBMC fractions of the same donor [27]. The here proposed system of long-term PBMC cultures for T cell evaluation is simple and robust and has the additional advantage that it enables the crosstalk between different types of immune cells.

We observed that the strong expansion of antigen-specific T cells took place after an initial lag phase of about seven days. The timing of this pronounced expansion is in line with other publications [33,34,35,36]. However, different from these studies, here we observed that expansion took place even in the absence of exogenous cytokines (i.e., IL-2 or IL-21). This is in agreement with previous studies highlighting the role of TCR signaling as a driving force for T cell proliferation [37]: TCR signaling leads to an increase in intracellular Ca^+2^ concentrations, which in turn triggers IL-2 production [38,39]. Though we have not formerly proven TCR engagement in the current study, it is tempting to speculate that the strong T cell stimulation elicited by WIV resulted in exponentially increasing secretion of IL-2 over time, which eventually allowed proliferation of the antigen-specific T cells [40]. Yet, the production of endogenous IL-2 still needs to be verified. In our in vitro system, the addition of exogenous cytokines thus appears unnecessary and might even reduce the discriminatory power of the system to distinguish between vaccines with different immune cell-stimulating capacities. Nevertheless, it is worthwhile to explore the advantages and disadvantages of cytokine addition in detail; cytokines might be necessary when the system is going to be employed for de novo antigens. Different from standard cytotoxic T lymphocyte (CTL) assays where T cell responses are measured five days after re-stimulation [41,42] using a 10-day long-term culture approach allowed us to assess vaccine-induced responses in vitro even though numbers of (pre-existing) antigen-specific cells were low initially. 

Our results demonstrate that WIV and split vaccines induced antigen-specific T cell responses of different magnitudes indicated by significantly higher amounts of total IFNγ produced in WIV-stimulated than in split vaccine-stimulated PBMC cultures. Similar results were found by Halbroth and colleagues who, using DC-T cell co-cultures, also observed an enhanced ability of WIV as compared to the split vaccine to elicit virus-specific CD8^+^ T cell responses [30]. These results probably reflect differences in the stimulatory properties of the two vaccines. Although both contain the same proteins, they differ in their physical characteristics: WIV consists of intact virus particles, whereas the split vaccine is composed mainly of protein aggregates or soluble proteins [8,11]. WIV particles are easily taken up by APCs due to their structure and “optimal antigen organization” [10]. Furthermore, WIV particles retain membrane fusion properties and can thus deliver influenza antigens directly into the cytosol from where they have access to the MHC I presentation pathway for presentation to CTLs [11]. Additionally, WIV is intrinsically adjuvanted with viral single-stranded RNA (ssRNA), which, through the triggering of TLR7 and TLR8, leads to activation of DCs [9,43]. In contrast, the split vaccine contains only minor amounts of ssRNA [43,44] (although there is some inconsistency about this point in the literature [45]) and therefore has a reduced capacity to activate DCs [30]. Overall, the higher immunogenicity of WIV over split observed in this study is in line with previous reports from in vivo studies. While an H5N1 WIV vaccine readily induced influenza-specific CD4^+^ T cells in human volunteers [46], split vaccine at the same antigen dose (2 × 7.5 µg HA) did not unless adjuvanted with AS03 [47]. In order to assess the sensitivity of our in vitro system, we additionally tested candidate influenza peptide vaccines, which differ from WIV and split vaccines, by comprising only two or four antigenic epitopes. In the long-term PBMC cultures, we could readily observe antigen-specific responses to the peptides, although these were of lower magnitude than those induced by WIV and split vaccines. These results underline the robustness of the developed in vitro system and the possibility to use it to assess the relative capacity of vaccine candidates to elicit T cell responses. 

Our results demonstrate that long term stimulation of PBMCs with WIV, split vaccine, or peptides generally induced recall responses; thus, it activated memory rather than naïve T cells. Although the vaccines we used were derived from the H5N1 virus (to which the blood donors were most likely naïve), this was according to expectations since the dominant T cell epitopes of influenza are mainly located in conserved areas of the internal viral proteins. CD4^+^ and CD8^+^ T cells responding to the vaccines mainly had an effector memory phenotype, followed by cells with a central memory phenotype. In accordance with our results, phase I clinical trials with split H5N1 vaccine or a vaccinia virus-based universal influenza vaccine candidate have also shown a prevalence of effector memory over central memory CD4^+^ T cell responses [48]. The predominance of effector CD4^+^ T cells was also observed when PBMCs from naïve individuals were stimulated in vitro with live H1N1pdm09 virus [49]. The same study reports that effector memory cells also prevailed among the influenza-specific CD8^+^ T cells, in line with our results. Other authors investigating influenza-specific CD8^+^ T cell responses in vivo or in human PBMCs also report on the predominance of CD8^+^ T_EM_ rather than T_CM_ [15,50,51,52,53]. 

Interestingly, we observed that exposure of long-term PBMC cultures to WIV or split vaccine, though not changing the overall frequency of T_FH_ cells, significantly enhanced the proportion of T_FH_ cells with an activated phenotype (ICOS^+^ and ICOS^+^IL21^+^). T_FH_ cells are required for the generation and maintenance of germinal center reactions in secondary lymphoid organs and the generation of high affinity antibodies [19,20]. Recently, CD4^+^CXCR5^+^ T cells were identified as the circulating counterpart of germinal center T_FH_ cells [21]. Moreover, the number of activated blood T_FH_ cells was found to correlate with the magnitude of newly generated T_FH_ cells in secondary organs [54], indicating that the analysis of blood T_FH_ subsets can help to get insight into ongoing T_FH_ responses [55]. Studies in influenza-vaccinated individuals revealed that the number of ICOS^+^ T_FH_ cells in blood peaked at day seven post-immunization and correlated with an increase of antibody titers and with the generation of high-avidity antibodies [23,24]. Activated circulating T_FH_ cells can thus serve as a biomarker for vaccination success [26]. Conversely, activation of antigen-specific T_FH_ cells in vaccine-exposed long-term PBMC cultures as observed by us can likely be considered as an indication for the capacity of a vaccine to stimulate T_FH_ cells and thus antibody production in vivo. Yet, parallel in vitro and in vivo studies are required to determine whether this is indeed the case.

CFSE dye dilution and/or intracellular staining for IFNγ have been used earlier to study T cell responses ex vivo in cultures of human PBMCs [56,57,58,59]. Yet, these studies mainly report on successful in vitro stimulation of T cells of recently vaccinated individuals. Other attempts to model the human immune system in vitro, for example, the MIMIC^®^ system, use approaches requiring separation and sorting of cell populations and/or 3D reconstructions of lymphoid organs (reviewed in [60]). These systems are highly sophisticated, yet, may be too cumbersome for many laboratories. In contrast, our in vitro system consists of cultures generated from readily available PBMCs of healthy, not recently vaccinated individuals. The system requires minimal manipulation of the cells, yet, proved suitable to compare and characterize (recall) T cell responses to different types of vaccines in depth. Our approach does not only allow the differentiation of responses between naïve T cells and different memory T cell subtypes but also to dissect T_FH_ responses. 

A limitation of our study is that it demonstrates the suitability of the developed in vitro system for the evaluation of T cell responses to previously encountered antigens but not to de novo antigens. Using influenza vaccines, we did observe only a minor response in the naïve T cell subset. Our study thus proves the potential usefulness of the system for evaluation of (novel) vaccines intended for boosting pre-existing immunity (e.g., against varicella, influenza, tetanus). Further experiments should address the feasibility of assessing naïve T cell responses by making use of antigens to which individuals are immunologically naïve. Preliminary experiments using Hepatitis B antigen imply that the described in vitro system does also allow T cell activation with novel antigens (Tapia Calle, unpublished observations). Yet, effective in vitro priming of naïve T cells will likely require further fine-tuning and optimization of the system, for example, by using suitable cytokines. Another possible limitation of our study and the described in vitro vaccine evaluation system is that it relies on the use of human PBMCs, which has often been associated with the issue of donor variability and thus inconsistencies in the results [61,62]. In our study, PBMCs of all donors responded in a similar way, although not always to the same extent, underlining that the system can deliver meaningful information despite donor variability. Furthermore, as mentioned above, an important issue will be to reveal whether responses to vaccines, as observed in the long-term PBMC cultures, correlate with responses the vaccines elicit in the donors in vivo. For such a direct validation, data from pre-vaccination PBMC samples will need to be compared to data from post-vaccination PBMC samples as well as antibody titers and T cell responses. Nevertheless, the correlation between the effectiveness of different vaccines to activate T cells in our in vitro system and in in vivo trials (according to published data) is a strong indication that the PBMC cultures deliver meaningful results.

## 5. Conclusions

The development of vaccines for clinical use is time-consuming and expensive since results obtained in animal experiments are often poorly predictive for vaccine performance in humans. Here we describe a model system consisting of long-term cultures of unfractionated human PBMCs for assessing T cell recall responses to vaccines in detail. The system allows for the comparison of the stimulatory capacity of different vaccine types, to identify responding T cell subpopulations (naive, T_CM_, T_EM_, T_EMRA_) and to determine their cytokine production profile and cytotoxic potential. In addition, the system is suitable for measuring vaccine effects on T follicular helper cells, which have been described in vaccines as an early correlate of humoral immune responses. Given these properties, the newly developed in vitro system can be used to gain insight into the effects and working mechanisms of candidate vaccines in a human immune cell context, so far for vaccines, including recall antigens, but in the future, hopefully also for de novo antigens. As such, the system is useful for the comparison of candidate vaccines and can assist in the selection of the most promising candidates for further clinical evaluation. 

## Appendix A

**Table A1:** Peptide’s details.

Epitope	Protein	Amino Acid Position	CD4/CD8	Length
LKREITFHGAKEIALSY	M	103–119	CD4	16
TYQRTRALVRTGMDPRM	NP	147–163	CD8	16
EALMEWLKTRPILSPLTK	M	40–57	CD8	17
RMCNILKGKFQTAAQRAM	NP	221–238	CD4	17

**Table A2:** Overview of antibodies used for flow cytometry.

Panel 1. Activation, Cytotoxicity and IFNγ of T Cells T Cells	Panel 2. T Cell Subsets and IFNγ	Panel 3. T Follicular Helper Cells
Live/Dead-Pacific Orange	Live/Dead - Pacific Orange	Live/Dead - Pacific Orange
CD3-Pacific Blue	CD3-Pacific Blue	CD3-Pacific Blue
CD4-APCCy7	CD4-APCCy7	CD4-APCCy7
CD8-PerCPCy5.5	CD8-PerCPCy5.5	ICOS-PerCPCy5.5
IFNγ-PeCy7	IFNγ-PeCy7	CXCR5-PeCy7
CD25-PE	CCR7-PE	IL21-PE
CD107-APC	CD45RO-APC	-
